# Gag-protease coevolution analyses define novel structural surfaces in the HIV-1 matrix and capsid involved in resistance to Protease Inhibitors

**DOI:** 10.1038/s41598-017-03260-4

**Published:** 2017-06-16

**Authors:** Francisco M Codoñer, Ruth Peña, Oscar Blanch-Lombarte, Esther Jimenez-Moyano, Maria Pino, Thomas Vollbrecht, Bonaventura Clotet, Javier Martinez-Picado, Rika Draenert, Julia G. Prado

**Affiliations:** 1 Lifesequencing SL, Paterna, Spain; 2 Universidad Catolica de Valencia, Valencia, Spain; 3 AIDS Research Institute IrsiCaixa, Hospital Universitari Germans Trias i Pujol, Universitat Autònoma de Barcelona, Badalona, Spain; 4 Veterans Affairs San Diego Healthcare System, San Diego, California, USA; 5 University of California San Diego, La Jolla, California, USA; 6 Universitat de Vic–Universitat Central de Catalunya, Vic, Spain; 7 Institució Catalana de Recerca i Estudis Avançats (ICREA), Barcelona, Spain; 8 Medizinische Poliklinik, Klinikum der Ludwig-Maximilians-Universität München, Munich, Germany

## Abstract

Despite the major role of Gag in establishing resistance of HIV-1 to protease inhibitors (PIs), very limited data are available on the total contribution of Gag residues to resistance to PIs. To identify in detail Gag residues and structural interfaces associated with the development of HIV-1 resistance to PIs, we traced viral evolution under the pressure of PIs using Gag-protease single genome sequencing and coevolution analysis of protein sequences in 4 patients treated with PIs over a 9-year period. We identified a total of 38 Gag residues correlated with the protease, 32 of which were outside Gag cleavage sites. These residues were distributed in 23 Gag-protease groups of coevolution, with the viral matrix and the capsid represented in 87% and 52% of the groups. In addition, we uncovered the distribution of Gag correlated residues in specific protein surfaces of the inner face of the viral matrix and at the Cyclophilin A binding loop of the capsid. In summary, our findings suggest a tight interdependency between Gag structural proteins and the protease during the development of resistance of HIV-1 to PIs.

## Introduction

The introduction of protease inhibitors (PIs) as part of the highly active antiretroviral therapy (HAART) have led to a dramatic reduction in morbidity and mortality rates in HIV-1–infected patients^1^. PIs have high intrinsic antiviral activity and are among the most potent antiretroviral drugs (ART) available in clinical practice to date. In fact, only simplification strategies with boosted PIs have proven to be as efficacious as triple ART in maintaining continuous virological suppression^2, 3^.

PIs target the active site of the HIV-1 protease (PR). Protease activity is essential for the generation of full infectious viral particles through the cleavage of Gag and Gag-pol polyproteins. Despite the high genetic barrier of the PI, the emergence of mutations at the protease active site leads to drug resistance. Mutations in HIV-1 causing resistance to PIs reduce the affinity of the drug for the active site. These mutations are generally followed by a stepwise accumulation of additional mutations in protease that partially rescue its activity^4^. Moreover, mutations in the Gag polyprotein at protease cleavage sites have generally been shown to contribute to resistance to PIs by restoring the interaction with the cleavage sites and compensating for defects in viral replicative capacity^5, 6^.

However, the above-mentioned “traditional” PI resistance pathways have been challenged by studies that evidence virological failure of PI-treated patients in the absence of PI resistance mutations^7^. Various studies have demonstrated the direct contribution of Gag mutations to drug susceptibility. Thus, mutations at Gag cleavage site positions A431V, K436E and I437V/T conferred resistance to PIs in the absence of drug resistance mutations at the active site of the protease^7–9^. Moreover, central residues of the Gag matrix (R76K, Y79F, and T81A) have been directly associated with reduced susceptibility to PIs and increased viral replicative capacity^10, 11^. These “alternative” PI resistance pathways have been shown to include mutations in the cytoplasmic tail of gp41 that can alter interactions between gp41 and Gag, thus affecting viral entry^12, 13^. The previous studies evidence the importance of HIV-1 Gag in the mechanisms of susceptibility to PIs and support the association of Gag and protease as a functional unit. Furthermore, these studies demonstrate that, despite many years since the introduction of PIs, the determinants of virological failure have not been fully characterized.

To gain new insights into the identification of novel determinants in Gag associated with resistance to PIs, we traced virus evolution by combining Gag-protease bulk and single genome sequencing with coevolution analysis of protein sequences in 4 patients treated with PIs over a 9-year period. Using this approach, we determined hotspots of protease coevolution under pressure from PIs in Gag structural proteins, mainly in the matrix, but also in the capsid. Moreover, 3-dimensional information on coevolving sites in the matrix and the capsid shed light on the structural and functional constraints governing Gag coevolution under pressure from PIs.

## Results

### HIV-1 mutations at Gag cleavage and non-cleavage sites emerge concomitantly *in vivo* during resistance to PIs

**Figure 1:**
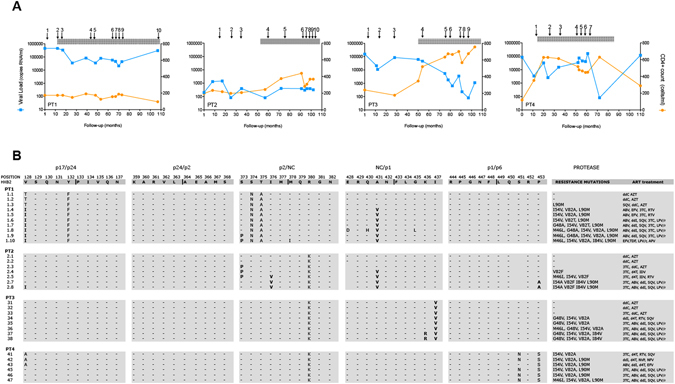
Gag cleavage sites and protease mutations in long-term PI-experienced HIV-1–infected patients. (**A**) Clinical follow-up of patients. Blue lines show HIV-1 viral load and orange lines show CD4+ T-cell counts. The grey bar on top represents time on antiretroviral treatment containing PIs. Arrows and numbers indicate the time points of the samples tested in the study. (**B**) HIV-1 Gag cleavage site mutations and PI resistance mutations. The top line shows the sequence numbering according to the HXB2 reference sequence for all HIV-1 Gag cleavage sites. Cleavage site mutations (CSM) are indicated by letters. Bold letters identify the CSM previously associated with exposure to PIs. Only PI resistance mutations in the protease are shown on the right column. AZT, zidovudine; 3TC, lamivudine, ddC, zalcitabine; ddI, didanosine; d4T, stavudine; EFV, efavirenz; TDF, tenofovir; ABC, abacavir; SQV, saquinavir; RTV, ritonavir; IDV, indinavir; LPV/r, lopinavir; NFV, nelfinavir; APV, amprenavir.

We first investigated mutational changes in HIV-1 during the development of resistance to PIs and their distribution across Gag and protease regions. We sequenced the HIV-1 Gag-protease coding region from longitudinal plasma samples in 4 patients (PT1 to PT4) over a 9-year period of antiretroviral treatment containing PIs (Fig. 1A). Sequence analysis confirmed the stepwise accumulation of Gag cleavage site mutations (CSM) at the following positions: V128I at p17/p24; S373P, I376V at p2/NC; and A431V NC/p1, K436R NC/p1 and P453A at p1/p6. Of these Gag CSM, A431V was previously associated with drug resistance mutations at positions M46I/L and V82A/T in the protease, K436R was associated with the mutation V82A, and P453A was associated with the drug resistance mutations I84V and L90M in the protease^4^. Indeed, CSM associated with drug resistance mutations in the protease have been previously associated with PI exposure *in vivo* and in some cases (V128I, A431V, K436R, P453A) with PI resistance^4^. Mutational evolution of the virus in all patients rendered virus multiresistant to all PIs over time, with the exception of darunavir (Stanford HIVdb Genotypic Resistance Interpretation Algorithm, data not shown).

However, the patterns and distribution of mutations in Gag varied between the patients. In PT1, we observed the emergence of HIV-1 Gag CSM at positions V128I (p17/p24) and A431V (NC/p1), together with the selection of the resistance mutations I54V and V82A in the protease during treatment with RTV (Fig. 1B). Moreover, the selection of S373P (p2/NC) in this patient matched the introduction of LPV/r. In the case of PT2, Gag CSM S373P (p2/NC) was present before the selection of A431V (NC/p1) and V82F in the protease after the introduction of IDV^4^. The presence of S373P before introduction of IDV could be associated with the polymorphic nature of this residue in treatment-naïve individuals^14^. Therefore, S373P could be selected in the absence of PIs, thus conferring a transitory advantage for the selection of protease drug resistance mutations, as suggested by the association of S373P to weaker virological responses to SQV/r^15^. The stepwise accumulation of I376V (p2/NC), V128I (p17/p24) and P453A (p1/p6) in PT2 supports the emergence of complex mutational patterns in the protease, including I54A, V82F, I84V and L90M. In contrast, for PT3, I437V was present, and the selection of K436R (NC/p1) in Gag CSM was concomitant with the emergence of the I84V mutation during treatment with LPV/r. No mutations were observed at the Gag cleavage sites in PT4 (Fig. 1B).

Next, we aimed to identify the regions in Gag with preferential accumulation of mutations derived from exposure to PIs. We performed a comparative analysis of Gag sequence evolution by comparison of bulk sequences before and after the introduction of PIs in each patient. We observed how amino acid variation concentrated in p2 (28.57%), followed by NC (14.55%), p17 (14.39%) and p6 (13.46%). We found very limited variation in p24 (2.61%) (Table 1). After the exclusion of Gag cleavage site positions from the analysis, sequence variation concentrated in p17 (12.12%) followed by NC (10.91%) and p6 (11.54%). We observed no differences in p24 (2.61%). The limited variation observed in p24 may be associated with the extreme genetic fragility of the protein to mutational changes^16^. Overall, these data indicate that both Gag CSM and non-CSM are required for the development of resistance to PIs by HIV-1.

**Table 1:** Amino acid variation in Gag proteins.

Gag	Length	Amino acid changes	Amino acid changes outside CS	Total variation (%)	Variation outside CS (%)
p17	132	19	16	14.39	12.12
p24	230	6	6	2.61	2.61
p2	14	4	1	28.57	7.14
NC	55	8	6	14.55	10.91
p1	16	1	0	6.25	0
p6	52	7	6	13.46	11.54

Amino acid changes were calculated as the median of individual changes per patient by direct comparison with the naïve PI sequence per each patient. (%) Frequency of variation was calculated as the number of amino acid changes in relation to protein length. CS, Cleavage sites

### HIV-1 Gag coevolving residues identify the matrix protein as the major contributor to protease evolution under selective pressure from PIs

We performed Coevolution Analyses for Protein Sequences (CAPS) to accurately identify correlated Gag residues involved in the evolution of the protease and resistance to PIs. This method has previously been applied in numerous case studies similar to ours^17–19^, and further detail on the methodology of CAPS analysis can be found in the Methods section. Thus, we analysed 171 HIV-1 Gag-protease sequences obtained by single-genome amplification (SGA) using CAPS. The sequences were equally distributed across patients and across the times from initiation of therapy with PIs (PT1, n = 44; PT2, n = 38; PT3, n = 41; and PT4, n = 48). We used SGA to avoid PCR resampling and recombination events, as previously reported^20^, and to accurately assess inter-protein mutational linkage, which is difficult to address in independent short reads^21, 22^.

**Figure 2:**
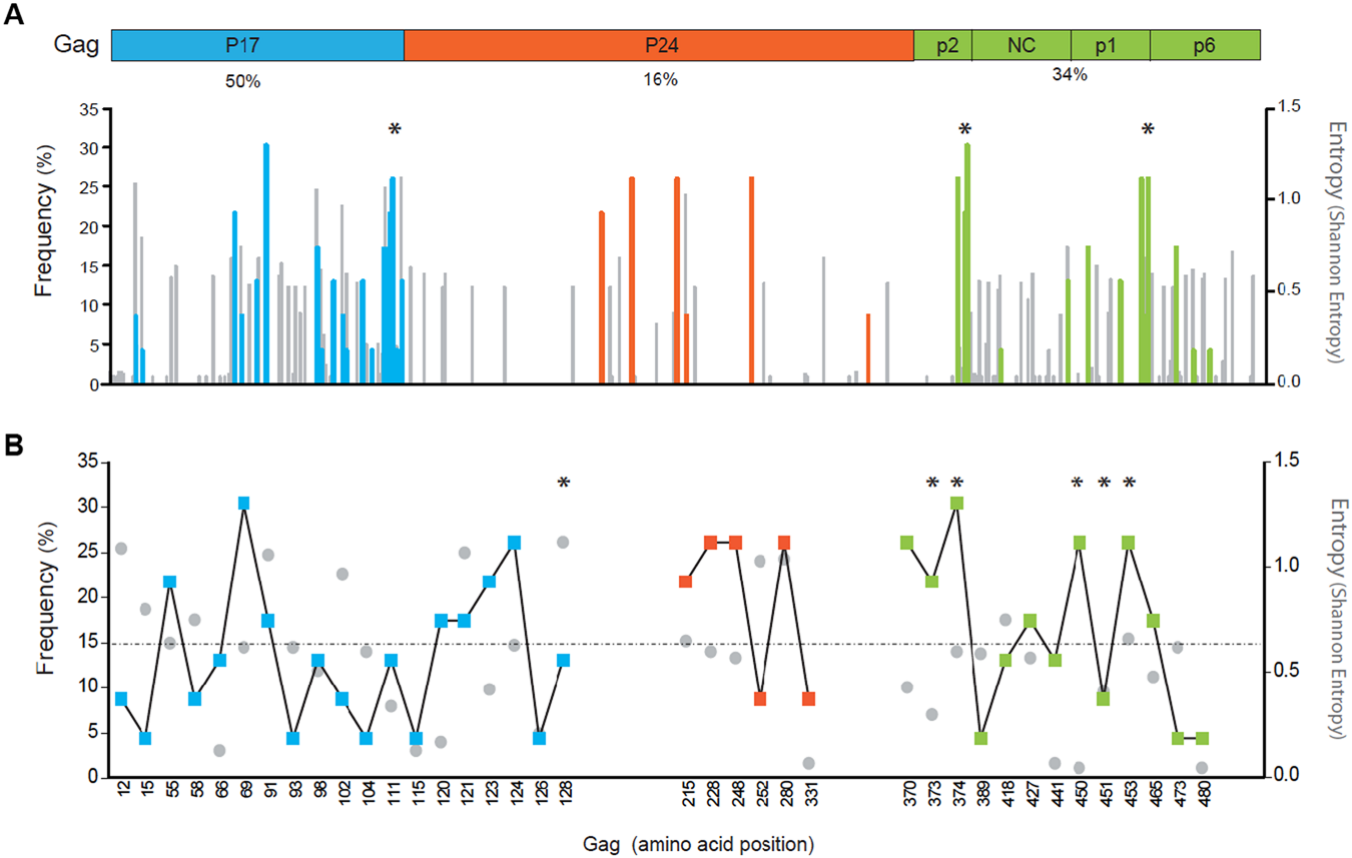
HIV-1 Gag correlated residues with protease. (**A**) Distribution of correlated residues in the Gag polyprotein. Left Y-axis coloured lines show the frequency of correlated residues in Gag among coevolving groups (%). Right Y-axis with grey lines shows the Shannon entropy of the correlated residues. Coloured lines indicate correlated residues; p17 in blue, p24 in orange, and p2, NC, p1 and p6 in green. (**B**) Specific residues of Gag correlated sites with the protease. Coloured squares indicate the frequency of the sites. Grey dots indicate the entropy of the sites. The line indicates the 15% frequency. Asterisks indicate residues at Gag cleavage sites. Numbering is according to the HXB2 reference sequence.

By using CAPS on our sequence dataset, we were able to capture previous correlations of Gag with the protease at cleavage site positions V128 (p17/p24), S373 and S374 (p2/NC), Q450, S451 and P453 (p1/p6) (Fig. 2)^4^, which we used as quality benchmark of our analytical tools. Moreover, our analyses established significant correlations with a total of 38 residues across Gag domains. Of those residues, 19 (50%) were located in the p17 viral matrix, 6 (16%) were located in the p24 viral capsid and 13 (34%) were distributed across p2/NC/p1/p6 proteins (Fig. 2A). In addition, 32 out of 38 residues were located outside Gag cleavage sites, thus, indicating the importance of Gag residues outside cleavage sites in protease coevolution. Further details on the specific residues are shown in Fig. 2B, including the entropy of the site and site frequency among Gag-protease coevolving groups. Of note, positions with a frequency over 15% in coevolving groups include residues 55, 69, 91, 120, 121, 123, and 124 in p17; residues 215, 228, 248 and 280 in p24; and residues 370, 373, 374, 450, 453, and 465 in p2/NC/p1/p6 (Fig. 2B). Thus, these data support the role of p17 as the major contributor to protease evolution in terms of the total number of Gag correlated residues and their frequency among coevolving groups. Moreover, the lack of association between residue frequency in coevolving groups and Shannon entropy at the site (r^2^ = 0.08, rho = −0.29, data not shown) rules out the potential linkage between site-preferential coevolution and site variability, thus suggesting a direct effect of pressure from PIs on Gag-protease coevolution.

### HIV-1 Gag coevolving residues define specific protein surfaces in the matrix and capsid

Next, we defined Gag-protease groups of coevolution and investigated their distribution at the protein 3-dimensional level. The 38 pairs of correlated residues organized in a total of 23 Gag-protease coevolving groups. These groups are defined by pairs of correlated residues sharing significant correlations among all pairs within the group.

**Figure 3:**
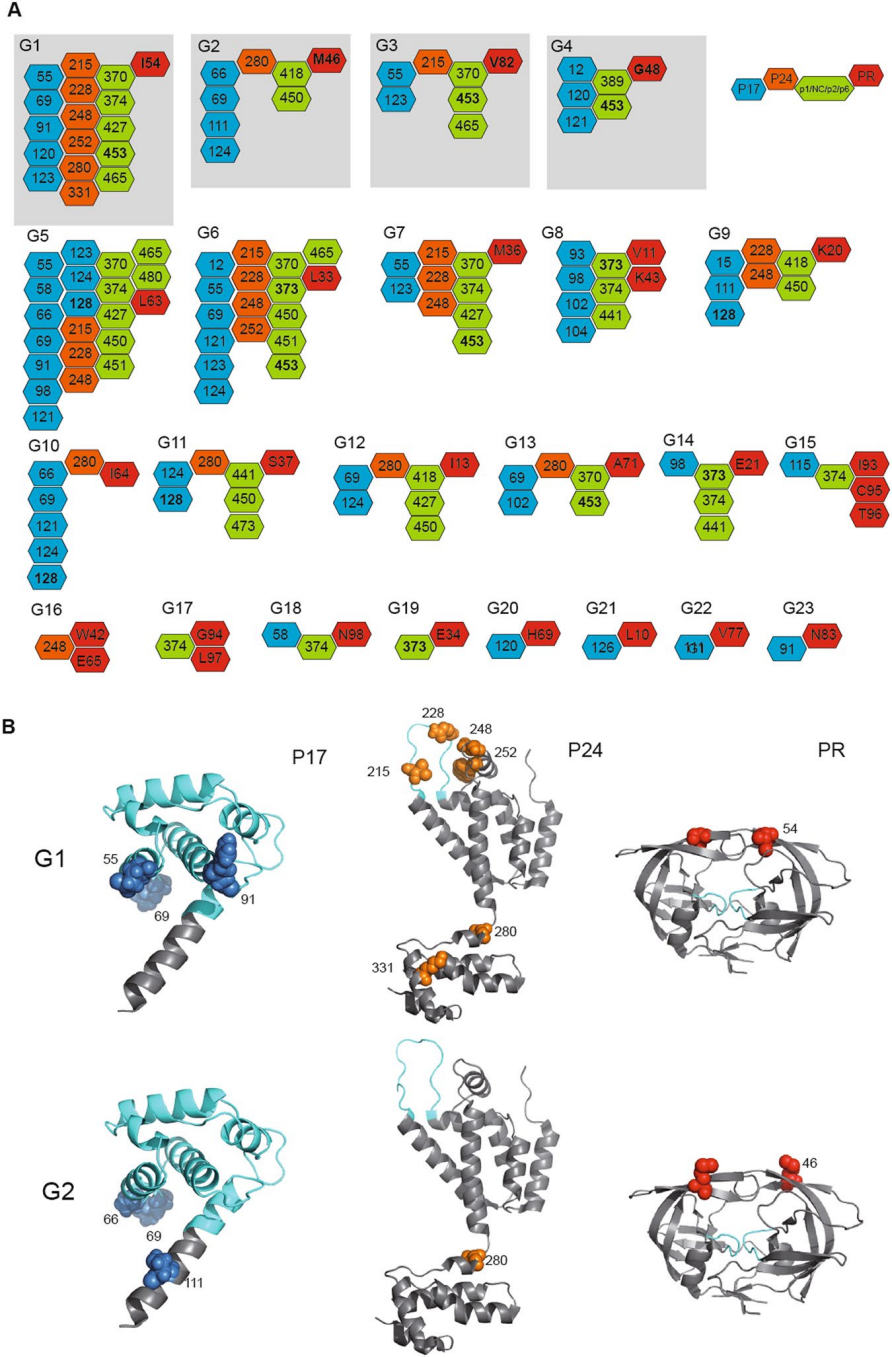
Coevolving groups of Gag-protease residues and protein structural mapping. (**A**) Coevolving Gag-protease groups. Protein residues within groups are indicated by coloured hexagons (blue for p17, orange for p24, and green for p1/NC/p2/p6). Grey squares indicate groups with protease residues associated with resistance of HIV-1 protease to PIs. Numbers in bold indicate positions previously associated with resistance and/or exposure to PIs. (**B**) Solved crystal structure of p17 (PDB 1HIW), p24 (PDB 1E6J) and protease (PDB 4dqf). Coloured spheres indicate coevolving residues in each structure blue for p17, orange for p24 and red for protease. The areas highlighted in cyan indicate; the globular domain in p17, the Cyp A binding loop in p24 and the enzyme active site in protease. Numbering is according to the HXB2 reference sequence. Coevolving residues represented in the p17 are limited to those present in the crystal structure.

The detailed composition of Gag-protease coevolving groups is shown in Fig. 3A. Groups of coevolution G1 to G4 included residues in the protease that were already associated with resistance to PIs (I54, M46, V82, and G48). In addition, G6, G8 and G23 included polymorphic sites in the protease (G6, L33; G8, V11 and K43; and G23, N83). Of note, 78% of the coevolving groups included two or more Gag domains and a single protease residue (Fig. 3A). Moreover, G20 to G23 had one residue in p17 (120, 126, 111 and 91) and one in the protease (69, 10, 77 and 83), further supporting coevolutionary constraints between p17 and the protease during treatment with PIs.

To further understand specific groups of coevolution, we performed an analysis of individual groups in previously solved crystal structures of p17, p24 and the protease. We selected G1 and G2, as they contain residues 54 and 46, which are directly associated with resistance to PIs. As shown in Fig. 3B, for G1 sites of coevolution between I54 and Gag include positions 55, 69, 91, 120 and 123 in p17 and 215, 228, 248, 252, 280 and 331 in p24. Residues 55, 92 and 91 in p17 are located in the globular domain. Indeed, residue 55 in helix 3, which is at the centre of the globular domain, interacts with helix 4 and is important for the structural stability of the protein^23^. By contrast, residues 69, 91 and 120 and 123, which are located at the α3/4 loop, 4/5 loop and helix 5, respectively, in p17, share properties of structural flexibility. Residues 215, 228, 248 and 252 in p24 are located at the Cyclophilin-A (CypA) binding loop or nearby. These residues are essential for binding with cellular CypA and the early events of HIV-1 replication^24^. Moreover, residues 280 and 331, which bridge the NTD and the CTD of p24, share properties of structural flexibility in the protein. Additionally, for G2 sites of coevolution between M46 and Gag include residues 66, 69, 111 and 124 in p17 and 280 in p24 (Fig. 3B). In this group, all Gag residues were located at regions of structural flexibility, which include the α3/4 loop (66, 69), helix 5 (111, 124) in p17 and the linker domain between NTD and CTD (280).

**Figure 4:**
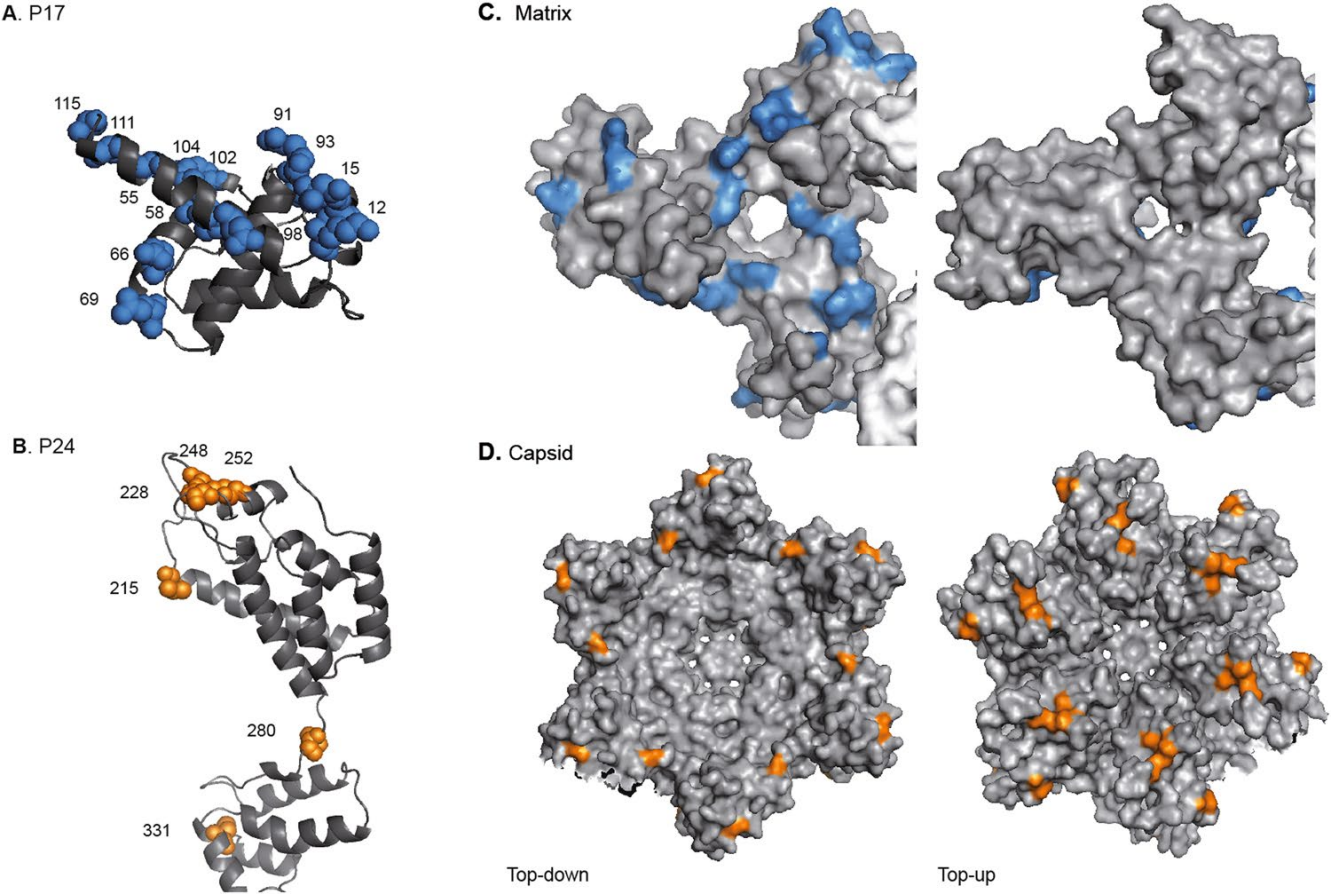
Mapping of coevolving HIV-1 p17 and p24 sites at the quaternary protein structural level. (**A**) Solved crystal structure of p17 (PDB 1HIW) and (**B**) p24 (PDB 1E6J). Coloured spheres indicate coevolving Gag residues in blue for p17 and in orange for p24. Numbering is according to the HXB2 reference sequence. (**C**) Matrix trimers with blue areas highlighting coevolving residues; (**D**) Capsid hexamers with orange areas highlighting coevolving residues. Structures on the left represent proteins seen top-down, and structures on the right represent proteins seen top-up. Coevolving residues represented in the p17 are limited to those present in the crystal structure.

To extrapolate these initial findings to a general model for HIV-1 Gag-protease coevolution, we pooled coevolving Gag residues and investigated their distribution at the protein structural level. Structural mapping of all the coevolving residues in p17 and p24 identified a specific distribution of correlated sites. Coevolving residues in p17 were distributed across helix 1 (12, 15), helix 3 (55, 58), the α3/4 loop (65, 69), the interconnection domain 4/5 loop (91, 93) and helix 5 (98, 102, 104, 111, 115) (Fig. 4A). Furthermore, coevolving positions in p24 defined clusters of residues in the CypA binding loop (215, 228) and its neighbouring helix 6 (248) and the  interconnection domain helix 6/7 (252). These findings support previous data demonstrating the contribution of Cyp-A binding loop mutations in compensating for fitness defects of PI-resistant viruses^25^. Moreover, only two coevolving residues in p24 were outside this region. Residues 280 and 331 at the interconnection domain of helix 7/8 and capping box of the helix 10 (Fig. 4B), which link the NTD and the CTD domains of p24, provide structural flexibility to the protein.

Similarly, at the quaternary protein structural level, the mapping of coevolving Gag residues revealed an organized spatial distribution (Fig. 4C,D). Thus, correlated residues in the matrix identified specific protein sectors or protein surfaces on the inner face of the trimer (Fig. 4C). The distribution suggests the presence of inter-protein interfaces among viral proteins. In contrast, the distribution of coevolving residues in the capsid at the CypA binding loop and nearby (Fig. 4D) on exposed surface areas may indicate functional constraints to regulate CypA incorporation in the virions. Overall, these data suggest specific structural and functional constraints in the matrix and the capsid driven by the development of protease resistance to PIs.

### Gag-protease coevolution during treatment with PIs is independent of HIV-1-specific CD8+T-cell immune pressure

The immunodominant nature of Gag in the generation of HIV-1–specific CD8+ T-cell responses argues for a potential contribution of cellular immune pressure to the evolution of the virus during treatment with PIs and detectable viral load. We measured cellular immune responses in PBMC using ELISpot with a panel of overlapping peptides (OLP) covering the Gag and protease regions to evaluate HIV-1 Gag evolutionary convergence between HIV-1–specific CD8+ T-cell immune pressure and drug pressure during the 9 years of treatment with PIs.

**Figure 5:**
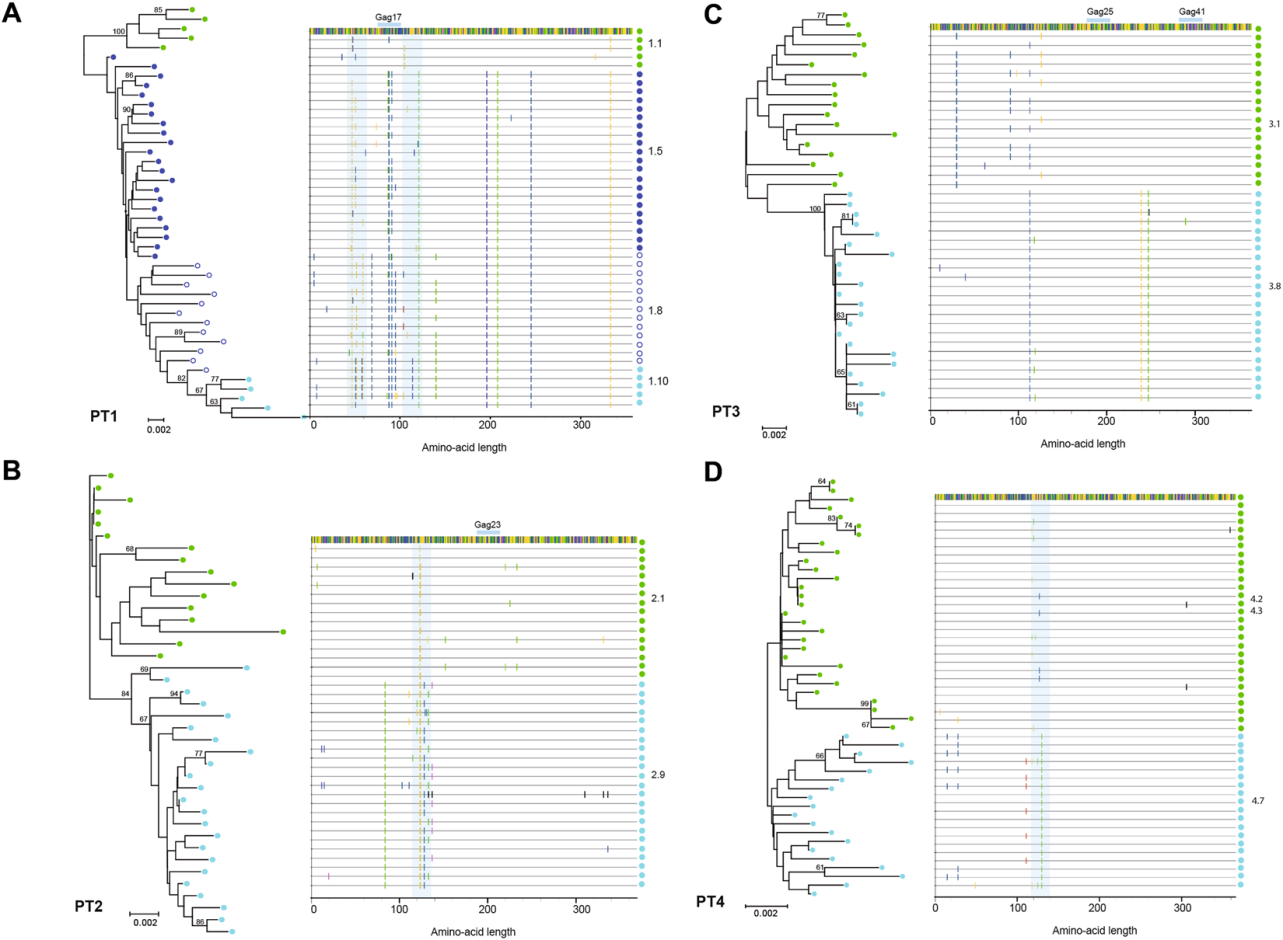
Correlated Gag resides and HIV-1 Gag-specific CD8+ T-cell responses. Neighbour-joining phylogenetic tree of Gag SGA nucleotide sequences represented for each study subject (**A–D**). Coloured dots represent SGA sequences from different time points, and numbers indicate sampling points. Bootstrap values above 60 are shown in the tree. The highlighted plots next to the tree represent Gag amino acid changes over time for each study subject by direct comparison with the earliest samples. Light blue shadows indicate Gag regions correlated with the protease, and blue lines on top of the highlighted plot show CD8+ T-cell responses to specific overlapping peptides in Gag.

In general, immune responses in the protease were almost absent, weak and focused on a few peptides in Gag (Gag overlapping peptides 17, 23, 25, and 41), with no major changes in breadth or magnitude during the follow-up period (Table S1). Additionally, in order to characterize in detail the convergence between HIV-1 evolutionary pathways derived from immunological pressure and treatment with PIs in Gag and protease, we merged immunological data with data obtained from the CAPS analysis for each patient. As shown in Fig. 5A–D, we did not find convergence between coevolving Gag sites obtained by CAPS and sites of CD8+ T-cell immune pressure. These data suggest an independent effect of PI-driven evolution of Gag and indicate no effect of immune pressure by CD8+ T cells on Gag-protease coevolution.

## Discussion

The potency of PIs, which results from their multi-step inhibitory mechanism, leads to an unusual complexity of the mutational pathways to HIV-1 resistance^12^. A direct reflection of this phenomenon is that most of the patients who experience virological failure with PIs nowadays do so in the absence of drug resistance mutations at the active site of the viral protease^8^. The complex mutational dynamics of HIV-1 under treatment with PIs is not fully understood. Therefore, a better understanding of the HIV-1 evolutionary pathways is crucial when attempting to identify novel determinants of virological failure during treatment with PIs. This information could be useful to build molecular models and improve drug design to ensure long-term treatment efficacy.

Over the last few years, various studies have revealed the major contribution of Gag to changes in susceptibility to PIs in the presence or absence of mutations in HIV-1 protease^7, 9–11, 26^. However, most of these studies rely on unique patient-derived viral genetic backgrounds and cross-sectional analyses of bulk sequences that cannot capture the complex dynamics of Gag-protease evolution under pressure from PIs. To overcome previous study limitations, we analysed longitudinal Gag-protease sequences over 9 years of PI-containing treatment in 4 patients. Moreover, we obtained Gag-protease sequences by SGA in order to avoid PCR artifacts and to accurately assess Gag-protease inter-mutational linkage by coevolution analysis. To our knowledge, no other experimental design has offered as much detail of Gag-protease mutational dynamics of HIV-1 evolution to identify Gag hotspots potentially involved in the development of resistance to PIs. Moreover, our data help to define Gag coevolution in the setting of pressure from PIs, thus excluding natural variation, common ancestors and the selective pressure from the immune system of this otherwise highly immunogenic region^27, 28^_._

Our results demonstrate that more than 50% of correlated Gag-protease residues lie in the viral matrix outside cleavage sites and are present in more than 85% of the coevolving groups. The most frequent correlated Gag residues in the matrix clustered around helix 3 (E55 and R58), the α3/4 loop (P66, and Q69) and helix 5 (K98, D102, E104, S111, and A115) in the p17 crystal structure. These data are consistent with the identification of frequent Gag-protease correlations within the first 200 residues in Gag^29^. In addition, out of the Gag correlated residues in the matrix, residues 55 and 58 located at helix 3 forms the globular core of the matrix through antiparallel contacts with helix 4, which is key for the structural stability of the protein. Mutations in the globular core of the matrix have been previously shown to reduce HIV-1 susceptibility to lopinavir^11^. Thus supporting the contribution of correlated sites in the matrix to HIV-1 evolution under pressure of PIs and potential development of resistance.

At higher protein structural levels, correlated residues in the matrix defined protein sectors on the inner face of the trimer. These surfaces, which were distributed across the tips and the centre of the trimer, identified potential novel inter-protein interfaces. In line with our findings, previous studies associated these surfaces with interactions between the C-terminal domain of the gp41 Env protein and the matrix. Indeed, Env proteins are incorporated into HIV-1 particles in a matrix trimerization-dependent manner^9, 30, 31^. Therefore, modifications in matrix stability could affect virus fusogenicity. Of the coevolving p17 residues identified in our study, three out of them (residues 66, 69 and 98) have been shown to contribute to matrix stabilization and incorporation of Env into immature viral particles^13, 30, 32^. Thus, we hypothesized that the viral matrix may affect resistance of HIV-1 to PIs disturbing particle maturation and virus fusogenicity. Our observations support recent findings that describe the impact of Env in resistance of HIV-1 to PIs^12^.

Together with correlated residues in the matrix, our study identifies a group of correlated Gag-protease residues in the capsid. These residues comprise only 6 positions of the longest Gag protein. Residues V215, M228, T248, N252, T280 and K331 were distributed across the NTD and CTD of the viral capsid. Positions V215, M228, T248, N252 and T280 clustered either in the CypA binding loop or nearby and organized in exposed surface areas of the capsid hexamer. The limited number of correlated residues in the capsid and their distribution restricted to exposed surface areas may be associated with the genetic fragility of the protein to mutational changes ^16^. Moreover, three of these positions (M228, T248, and N252) have previously been shown to compensate for fitness defects of p24 escape mutants driven by HIV-1–specific CD8+ T-cell responses^33, 34^. In addition, mutations in the CypA at position 219 have been shown to increase HIV-1 replication of PI-resistant variants^25, 35^. Thus, pointing towards evolutionary constraints in the capsid to modulate viral replicative capacity.

Our study provides novel insights into the contribution of Gag to protease evolution during the development of resistance of HIV-1 to PIs in two ways. We identify previously unknown residues and clusters of residues in Gag that are associated with the selection of PI resistance mutations in the protease (Supplementary Table 2). Moreover, our data demonstrate the role of the matrix as a hotspot of protease coevolution and pinpoint novel protein sectors in the matrix and the capsid that are associated with protease evolution under pressure from PIs. These findings may serve as a basis for future research into the potential mechanism of HIV-1 resistance to PIs of these regions.

Our study is subject to a series of limitations. First, we studied only four subjects with backbone treatments containing reverse transcriptase inhibitors (RTI) and switches of PIs over time. Therefore, we cannot completely exclude the influence by RTI backbone treatment on subsequent viral evolution. However, recent data suggests that there are minor genetic interactions between the RT and the Protease coding regions^36^ and  we can assume a minimal effect of RTI treatment in Gag-protease coevolutionary events. Second, we cannot exclude unknown interactions in immature Gag-pol polyproteins^37^, as structural mapping was performed in the mature protein crystal structures. Third, we did not perform direct measurements of viral susceptibility for PIs, and additional mutational, functional and structural studies of Gag correlated residues would be needed before conclusions can be drawn.

In summary, our study improves our understanding of Gag-protease dynamics of coevolution associated with HIV-1 constraints under pressure from PIs. In addition, our data underline the contribution of Gag structural proteins and identify novel residues and specific protein sectors that are potentially involved in the development of resistance of HIV-1 to PIs. Coevolution analysis of HIV-1 proteins when combined with those of additional functional and structural studies, may serve as a roadmap to guide the development of novel small molecules to increase treatment efficacy against HIV-1.

## Methods

### Study subjects

We studied 4 patients with chronic HIV-1 infection (PT1, PT2, PT3 and PT4) in whom resistance to PIs had evolved over a median of 4 years after initiation of treatment with PIs. The clinical parameters (median [IQR] for each of the patients) were as follows: PT1, 122 (39–158) CD4+ T cells/mm^3^; 69,000 (21,000–450,000) HIV-RNA copies/ml; PT2, 389 (80–1,460) CD4+ T cells/mm^3^; 457 (169–5,500) HIV-RNA copies/ml; PT3, 635 (177–757) CD4+ T cells/mm^3^; 5,500 (80–154,000) HIV-RNA copies/ml; PT4, 471 (118–630) CD4+ T cells/mm^3^; 37,500 (80–160,160) HIV-RNA copies/ml. Longitudinal plasma and peripheral blood mononuclear cell (PBMC) samples were available for all 4 patients (Fig. 1A).

### Ethics Statement

All methods and experimental protocols were approved by the Ethics Committee of Hospital Germans Trias i Pujol. The patients provided their written informed consent to participate in the study. The study was conducted according to the principles expressed in the Declaration of Helsinki.

### Bulk amplification of the HIV-1 Gag-protease coding region

Total viral RNA was extracted from longitudinal plasma samples with the QIAamp viral RNA mini kit (QIAGEN). Plasma samples with viral loads below 10,000 copies/ml, were concentrated at 23,000 *g* for 90 minutes at 4 °C by ultracentrifugation. Viral RNA was amplified by RT-PCR (Superscript One-Step, Thermo Fisher, Spain) with the following primer sets: MMOP3 5-AATCTCTAGCAGTGGCGCCCGAAC-3 (623-647_HXB2_) and MMOP5 5′-TAACCCTGCAGGATGTGGTATTCC-3′ (2849-2826_HXB2_). The amplification conditions were as follows: 30 minutes at 50 °C, 2 minutes at 94 °C, 15 seconds at 94 °C, 30 seconds at 53 °C, and 3 minutes at 68 °C for 40 cycles. A second polymerization step was carried out to amplify the Gag-protease coding region using a nested PCR (Platinum Taq DNA polymerase, Thermo Fisher, Spain) with the specific primers BssHII 5′-TTGCTGAAGCGCGCACGGCAAG-3′ (703-724_HXB2_) and ClaIR 5′-GGTACAGTATCGATAGGACTAATGGG-3′ (2575-2550_HXB2_). The PCR conditions were as follows: 30 seconds at 94 °C, 30 seconds at 94 °C, 30 seconds at 50/52 °C and 2 minutes 68 °C for 40 cycles. PCR reactions were purified using Exo-sap to remove primers dimers and reagents at 37 °C for 5 minutes and inactivated at 80 °C for 15 minutes.

### Single genome amplification (SGA) of the HIV-1 Gag -protease coding region

Extracted total viral RNA was reverse-transcribed to single-strand cDNA using Superscript III Reverse Transcriptase (Thermo Fisher, Spain) with the primer MMOP5 5′-TAACCCTGCAGGATGTGGTATTCC-3′ (2849-2826_HXB2_), as previously described^20^. The cDNA was used immediately for PCR or stored frozen at −80 °C. The cDNA was serially diluted in water with herring sperm DNA solution at 1,000 ng/ml to increase reproducibility at the dilutions used for PCR amplification and underwent sets of PCR reactions with the primers as described in the previous section. The SGA cut-off value for positive wells was 30%, as described elsewhere^20^. Positive PCR reactions were purified by Exo-sap to remove primer-dimers and reagents at 37 °C for 5 minutes and inactivated at 80 °C for 15 minutes.

### Sequence analyses

All sequences were obtained using Sanger reactions with the Macrogen service (Netherlands) and analyzed with Sequencher (Genecodes V). Sequences were aligned to HXB2 using the Bioedit package. Phylogenetic trees of bulk and SGA nucleotide sequences were constructed using the neighbour-joining phylogenetic method with 1,000 bootstrap replicates (MEGA version 5)^38^. Phylogenetic trees validated sequence specificity and absence of sample cross-contamination (Supplementary Figure 1). The Stanford University HIV Drug Resistance Database was used to identify protease drug resistance mutations. Drug resistance was interpreted based on mutation scoring (http://hivdb.stanford.edu/ web [accessed on August 2016]). Longitudinal SGA sequences were examined for amino acid variation over time using the highlighter program available at “https://www.hiv.lanl.gov”.

### Gag-protease coevolutionary analysis for protein sequences: CAPS

A total of 171 Gag-protease SGA sequences were aligned to the HXB2 reference sequence. SGA sequences were equally distributed across patients (PT1, n = 44; PT2, n = 38; PT3, n = 41; and PT4, n = 48) and follow-up points (before treatment with PIs and at the last time point available after introduction of PIs). We analysed total and patient-specific Gag-protease coevolution using a parametric model to identify coevolution events in protein-coding genes^39^. The model was implemented using the program Coevolution Analysis for Protein Sequences (CAPS)^40^. Briefly, CAPS compares the correlated variance of the evolutionary rates of pairs of amino acid sites, in a protein alignment adjusted for the time since the divergence of the 2 sequences they belong to. This method compares the transition probability scores between pairs of sequences at 2 particular sites, using the blocks substitution matrix (BLOSUM)^41^. The BLOSUM matrix applied in CAPS for each protein alignment applied depend of average sequence identity as previously described^39^. The significance of the CAPS correlation values was assessed by randomization of pairs of sites in the alignment, calculation of their correlation values, and comparison of the real values with the distribution of 10,000 randomly sampled values. Statistical significance was set at < 0.01. In order to correct for multiple tests for non-independence of data, CAPS implemented the step-down permutation procedure in both methods and corrected the probabilities. Gag-protease coevolution groups were constructed as previously indicated^39, 40^. In brief, we analysed significantly correlated pairs (p < 0.01) to identify clusters of amino acids sharing significant correlations among all the pairs and included those in a coevolving group (e.g. if 17 correlates with 23, 17 correlates with 35, 17 correlates with 82 and 23 correlates with 82; then, 17, 23 and 82 form a coevolving group).

Entropy per site was calculated in SGA sequences using the entropy tool (Entropy-One) from the Los Alamos database.

### Molecular modelling

Protein structures were obtained from RCSPDB—p24 (PDB 1E6J); p24 hexamer (PDB 3P05); p17 trimer (PDB 1HIW) and, protease (PDB 4dqf)—which are available at http://www.rcsb.org/pdb/home/home.do. The structures represent the B clade virus and were modified to show Gag correlated sites with protease using the PyMOL Molecular Graphics System, Version 1.8 (Schrödinger).

### HLA typing and assessment of HIV-1–specific CD8 + T-cell responses

High-resolution HLA class I typing for alleles A, B and Cw was performed using sequence-based typing methods. HIV-1–specific immune responses were assessed over time (Table S1) in cryopreserved PBMCs. In brief, after thawing, PBMCs were incubated with overlapping peptides covering the regions of HIV-1 Gag (48 peptides corresponding to the sequence HIV-1 SF2, 15-mers overlapping by 5 amino acids; CFAR/NIBSC, England) and protease (21 peptides corresponding to the consensus sequence of HIV-1 2001 for clade B, adapted 15-mers overlapping by 10 amino acids)^42^. Immune responses were measured using the interferon-γ (IFN-γ) ELISpot assay^43^. Wells were considered positive if they contained at least 50 spot-forming cells per 10^6^ PBMCs or 3 times above the background level (2 × mean + 3 × STD). Assays were performed according to availability of cells. It was not possible to test all peptides for all patients and all time points.

## Electronic supplementary material

Supplementary info
