# Supplementary material for: Gag-protease coevolution analyses define novel structural surfaces in the HIV-1 matrix and capsid involved in resistance to Protease Inhibitors
Source: Sci Rep. 2017 Jun 16;7:3717. doi: 10.1038/s41598-017-03260-4 (PMC5473930; doi:10.1038/s41598-017-03260-4)
Supplement: Supplementary file 1 — Supplementary info [file 41598_2017_3260_MOESM1_ESM.pdf]

Gag-protease coevolution analyses define novel structural surfaces in the HIV-1 matrix and capsid involved in resistance to Protease Inhibitors. Francisco M Codoñer, Ruth Peña, Oscar Blanch-Lombarte, Esther Jimenez-Moyano, Maria Pino, Thomas Vollbrecht, Bonaventura Clotet, Javier Martinez-Picado, Rika Draenert and Julia G. Prado

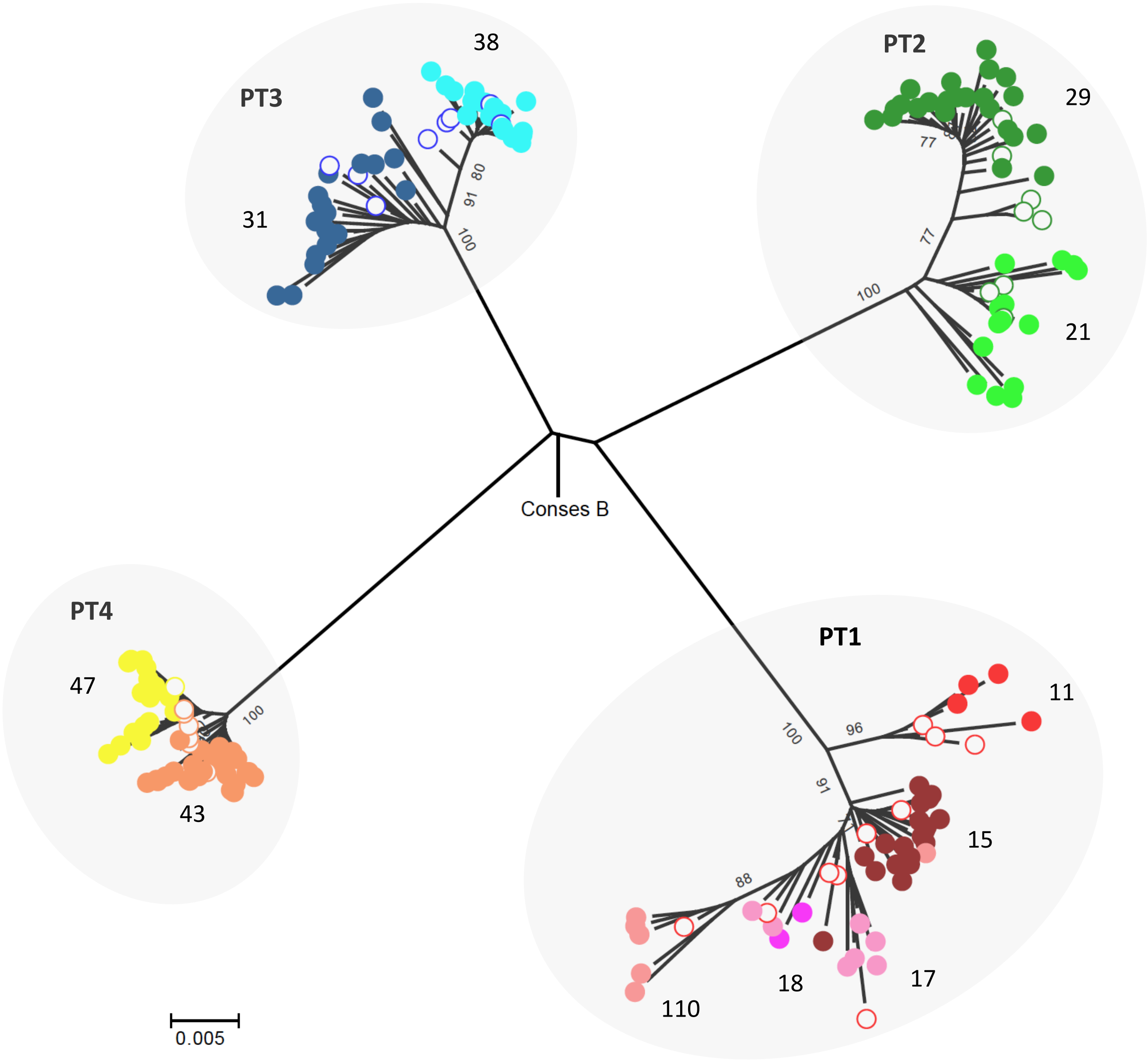

Figure S1. **Gag-protease neighbour-joining phylogenetic tree of bulk and SGA sequences from study subjects**. Bulk sequences are shown as empty dots and SGA sequences as coloured dots. Numbers indicate the sampling points. The tree is rooted to the consensus B sequence (Conses B) as an out-group. Only bootstrap values over 70 are shown.

Gag-protease coevolution analyses define novel structural surfaces in the HIV-1 matrix and capsid involved in resistance to Protease Inhibitors. Francisco M Codoñer, Ruth Peña, Oscar Blanch-Lombarte, Esther Jimenez-Moyano, Maria Pino, Thomas Vollbrecht, Bonaventura Clotet, Javier Martinez-Picado, Rika Draenert and Julia G. Prado

Table.S1 Gag CD8+ T-cell responses in long-term PI-experience patients

Subject	HLA class I molecules	Time-point	OLP	Sequence	Magnitude (SFC/M PBMCs)
PT1	A*02:05/23:01, B*08:01/49:01, C*07:01/07:01	1	none		-
		6	none		-
		8	Gag 17	RIDVKDTKEALEKIE	60
		10	none		-
PT2	A*02:01/02:05, B*44:03/49:01, C*07:01/16:02	1	Gag23	AFSPEVIPMFSALSEGA	80
		2	Gag23	AFSPEVIPMFSALSEGA	100
		4	Gag23	AFSPEVIPMFSALSEGA	110
		10	Gag23	AFSPEVIPMFSALSEGA	490
PT3	A*32:01/33:01, B*07:02/14:02, C*07:02/08:02	1	Gag 25	GATPQDLNTMLNTVGGH	10
		2	Gag 25	GATPQDLNTMLNTVGGH	170
			Gag 41	YVDRFYKTLRAEQASQEV	140
		3	Gag 25	GATPQDLNTMLNTVGGH	240
		4	Gag 25	GATPQDLNTMLNTVGGH	160
		8	Gag 25	GATPQDLNTMLNTVGGH	60
			Gag 41	YVDRFYKTLRAEQASQEV	130
PT4	A*01:01/23:01, B*08:01/44:03, C*07:01/04:01	NA	NA		NA

OLP; overlapping peptide. SFC/M; spot-forming cells-/million PBMCs, NA; not available

Gag-protease coevolution analyses define novel structural surfaces in the HIV-1 matrix and capsid involved in resistance to Protease Inhibitors. Francisco M Codoñer, Ruth Peña, Oscar Blanch-Lombarte, Esther Jimenez-Moyano, Maria Pino, Thomas Vollbrecht, Bonaventura Clotet, Javier Martinez-Picado, Rika Draenert and Julia G. Prado

Table.S2 Gag coevolving residues with the HIV-1 protease under PI pressure

Residue in Gag	Protein	Domain	Function	Mutations associate with PI resistance	Association with PI exposure		PI resistance	Experimental evidence
					In vivo	*In vitro*		
12	p17	Helix 1	targeting menbrane binding	E12K	unknown	yes	unknown	
15	p17	Helix 1	targeting menbrane binding	unknown	unknown	unknown	unknown	
55	p17	Helix 3		unknown	unknown	unknown	unknown	
58	p17	Helix 3		unknown	unknown	unknown	unknown	
66	p17	α3/4 loop		unknown	unknown	unknown	unknown	
69	p17	α3/4 loop		unknown	unknown	unknown	unknown	
91	p17	loop Helix 4/5		unknown	unknown	unknown	unknown	
93	p17	loop Helix 4/5		unknown	unknown	unknown	unknown	
98	p17	Helix 5		unknown	unknown	unknown	unknown	
102	p17	Helix 5		unknown	unknown	unknown	unknown	
104	p17	Helix 5		unknown	unknown	unknown	unknown	
111	p17	Helix 5		unknown	unknown	unknown	unknown	
115	p17	Helix 5		unknown	unknown	unknown	unknown	
120	p17	Helix 5		unknown	unknown	unknown	unknown	
121	p17	No crystal		unknown	unknown	unknown	unknown	
123	p17	No crystal		unknown	unknown	unknown	unknown	
124	p17	No crystal		unknown	unknown	unknown	unknown	
126	p17	No crystal		unknown	unknown	unknown	unknown	
128	p17	cleavage site p17/p24	recogniton for protease cleavage	V128I/T/A	yes	yes	yes	Callebaut et al, Antivir Ther 200[illegible]
215	p24	Cyclophiln A biding loop	binding to cellular CypA	unknown	unknown	unknown	unknown	
228	p24	Cyclophiln A biding loop	binding to cellular CypA	unknown	unknown	unknown	unknown	
248	p24	Helix 6		unknown	unknown	unknown	unknown	
252	p24	loop Helix 6/7		unknown	unknown	unknown	unknown	
280	p24	loop Helix 7/8		unknown	unknown	unknown	unknown	
331	p24	Helix 10		unknown	unknown	unknown	unknown	
370	p2			V370A/M/del	yes	unknown	unknown	
373	p2	cleavage site p17/p24	recogniton for protease cleavage	S373P/Q/T	yes	unknown	unknown	
374	p2	cleavage site p17/p24	recogniton for protease cleavage	A373P/S	yes	unknown	unknown	
389	NC			I389T	yes	unknown	unknown	
418	NC	Zn finger motif	RNA binding	unknown	unknown	unknown	unknown	
427	NC			unknown	unknown	unknown	unknown	
441	p1			unknown	unknown	unknown	unknown	
450	p6	cleavage site p1/p6	recogniton for protease cleavage	unknown	unknown	unknown	unknown	
451	p6	cleavage site p1/p6	recogniton for protease cleavage	S451T/g/R	yes	unknown	unknown	
453	p6	cleavage site p1/p6	recogniton for protease cleavage	P453L/A/T	yes	yes	yes	Kolli et al, J Virol 2009<br>Maguire et al, J Virol 2002
465	p6			unknown	unknown	unknown	unknown	
473	p6			unknown	unknown	unknown	unknown	
480	p6			unknown	unknown	unknown	unknown	
